# Evaluation of Decentralized Verifiable Credentials to Authenticate Authorized Trading Partners and Verify Drug Provenance

**DOI:** 10.30953/bhty.v4.168

**Published:** 2021-03-11

**Authors:** Ghada L. Ashkar, Kalpan S. Patel, Josenor de Jesus, Nikkhil Vinnakota, Natalie Helms, Will Jack, William Chien, Ben Taylor

**Affiliations:** 1 UCLA Health, Los Angeles, CA, USA; 2 Amgen, Thousand Oaks, CA, USA; 3 LedgerDomain, Las Vegas, NV, USA

**Keywords:** verifiable credentials, identity, DSCSA, pharmaceutical supply chain, drug verification

## Abstract

**Summary:**

In 2013, the Drug Supply Chain Security Act (DSCSA) was signed into law to address the growing threat of counterfeit drugs and to ensure prescription drugs remain safe and effective for patients. As part of this law, US pharmaceutical supply chain stakeholders are required to confirm the authorized status of trading partners for transactions and information disclosures, even when there is no prior business relationship. While larger Authorized Trading Partners (ATPs) have connectivity solutions in place, newer and smaller ATPs have not traditionally participated, including tens of thousands of dispensers. To unlock the full potential of the interoperable system mandated by the DSCSA, the authors tested eXtended ATP (XATP), a blockchain-backed framework for ATP authentication and enhanced verification in a real-world pharmacy with genuine drug packages. The objective of this research study was to prove that electronic authentication and enhanced verification can be achieved between ATPs using a mobile-based solution. Moreover, we tested accurate reading of drug and associated electronic med guides, flagging of expired and recalled drugs, and correct generation of documentation to support saleable returns.

**Methods:**

This study involved two dispensers and three participating manufacturers. Dispensers were onboarded to a mobile application and used supporting documentation to authenticate their identities, and then scanned 2D drug barcodes to submit drug verification requests to manufacturers (including 11 additional, randomly selected manufacturers). Genuine and synthetic drug package barcodes were used to test workflows against genuine and synthetic manufacturer serialization data records. Manufacturers authenticated the identity of requesting dispensers with verifiable credentials and responded to verification requests.

**Results:**

Enhanced drug verification was achieved, with 100% of requests successfully delivered to participating manufacturers and 88% of requests being delivered to other manufacturers (based on the pharmacist selection of random packages from the pharmacy). Drug verification matching against synthetic serialization data records resulted in 86% accuracy, with the 14% error rate attributed to human factors. All barcodes were successfully scanned and provided package-accurate data, and 97% of randomly selected packages successfully generated drug package inserts. All synthetic recalls and expired drugs were successfully flagged. Four of the manufacturers contacted were among the top 15 pharmaceutical manufacturers globally; all four responded.

**Conclusions:**

The XATP framework provides a secure, reliable, and seamless remote method to conduct enhanced verification as required by law. Interoperability between manufacturers and dispensers with no prior business relationship can be achieved on ‘day zero’ using mobile devices that enable digital authentication and rapid barcode scanning. As users retain control of their own private keys, the framework also mitigates the single-point-of-attack risks associated with centrally managed systems.

Over the past two decades, globalization and technological innovation have profoundly changed the US pharmaceutical supply chain, and thus, stakeholders face new and emerging requirements under the Drug Supply Chain Security Act (DSCSA) (1). One such requirement is an example of a ‘know your customer’ (KYC) requirement (2), in which each Authorized Trading Partner (ATP) (3) is required to confirm that their trading partner is also authorized (4).

As a result, tens of thousands of ATPs are responsible for authenticating each other’s identities before they can transact with one another, or even share certain information – even when there is no prior direct business relationship. While Verification Router Services (VRS) (5) have served to handle drug verifications for saleable returns as required under DSCSA (6), trading partner identity and status authentication remain a missing piece of the puzzle, especially for the broader community of small trading partners (7).

To address this challenge, the authors workshopped and tested a framework for ATP authentication, verification routing, and saleable returns documentation (8). Previously, a working group with representatives from the manufacturing and dispensing sectors found that this framework was capable of onboarding entities and their representatives, accrediting their licenses, and allowing them to share information with unique verifiable credentials (9). The study outlined in this article took the framework out of the virtual conference room and into a real-world production environment.

Under this framework, a dispenser with an iPhone and acceptable form of ID can be remotely authenticated as an ATP, can scan the 2D barcode from a serialized drug in their hand (10, 11), and can use an iOS app to send a verification request (12). This request, which pulls the drug’s GS1 Serialized Global Trade Item Number (SGTIN) (13, 14) from the scan, is used to identify the appropriate point of contact (POC) for the manufacturer or repackager. An email is sent asking the POC for validating each scanned drug against its master serialization record. The response can be used to generate supporting documentation to another ATP for a transaction (such as a saleable return (15)).

## Technical specifications

The XATP framework consists of five major components:

1. passwordless frontend mobile phone application (also called XATP),
2. application framework encompassing smart contracts and application logic (DocuSeal),
3. notification and verification service (Oraculous),
4. blockchain application server (Selvedge), and
5. backend blockchain (Hyperledger Fabric).

Users generate and hold their own private keys, and master National Drug Code (NDC) data are held locally on the client. Leveraging prior work with UCLA Health and Biogen (16), the Oraculous Interoperability Service unlocks interoperability between existing relational database management systems and hosted nodes of the distributed ledger (17). In this way, verification requests can be submitted, routed, and processed without the need for verifying organizations to provision their own nodes.

The framework leverages proven third-party services, including Splunk (analytics), Branch (mobile link service), OneSignal (push notifications), and Mailgun (email service). Cloud hosting and processing were achieved with LevelDB, Docker, Amazon EC2, Amazon Web Services, and MinIO. The Selvedge blockchain server was built with Golang on top of open-source Hyperledger Fabric 1.4 (Linux Foundation) components (18). Sealed documentation, private metadata, and Product Verification Certificates were held in private storage using MinIO, and public hash records were kept on the blockchain (8).

## Objectives

The earlier work of the XATP Pilot Group was conducted remotely using synthetic data in a closed environment. The objective of this study described in this article was to test the application framework in a real-world setting (the UCLA Specialty Pharmacy) using genuine drug packages and manufacturers’ production serialization data records.

Specifically, the objectives of this study were to test the following:

1. accurate reading of drug and associated electronic med guides,
2. expired and recalled drug flagging functionality,
3. authentication of a verification request with a verifiable credential, and
4. enhanced verification between dispensers and manufacturers.

## Methods and findings

This study included two rounds of testing with three sets of participants: dispensers, participating manufacturers, and other manufacturers based on packages randomly selected from the pharmacy (Table 1).

**Table 1:** Overview of study participant groups

Group	Members	Location
Dispensers	Pharmacy workgroup consisting of one pharmacist-in-charge (PIC) and two pharmacists (dubbed ‘POAs’, as they are designated through Power of Attorney to act on behalf of the PIC for the purposes of day-to-day operations)	UCLA Specialty Pharmacy
Participating manufacturers	Members of three pharmaceutical manufacturer organizations (among the top 15 pharmaceutical manufacturers globally) who participated in Zoom tests	Remote
Randomly selected manufacturers	Members of 11 pharmaceutical manufacturer organizations based on drug packages selected randomly from pharmacy inventory by the pharmacist	Remote

Dispensers fulfilled their role using the XATP application and iOS email clients, while manufacturers used platform-agnostic email clients and web interfaces. The dispensers and participating manufacturers used Zoom for real-time communication.

**Fig. 1:**
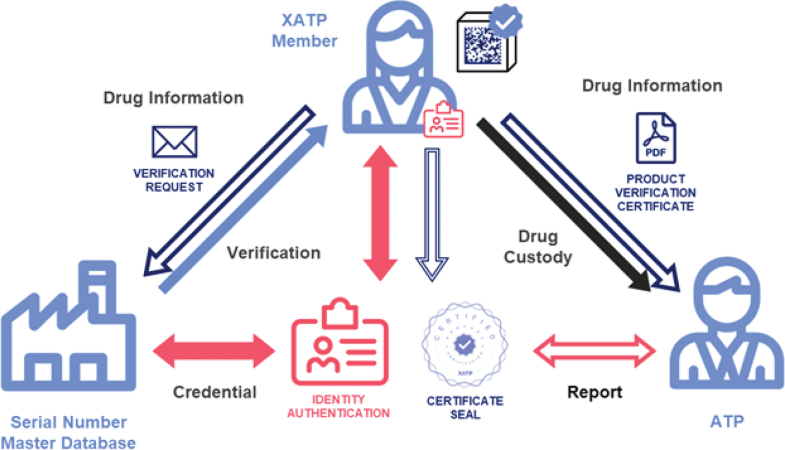
An overview of the XATP identity framework and enhanced verification routing. After authenticating his or her identity with an independent external validator, the dispenser can scan 2D barcodes on drug packages using an iOS app and submit verification requests as part of the saleable returns process. The package labeler (manufacturer or repackager) receives an email with a verifiable credential and buttons that link to secure Oraculous endpoints, allowing him or her to indicate that a drug is verified or unverified. This verification can be used to generate Product Verification Certificates that can be shared with, and independently authenticated by, other ATPs.

Prior to submitting verification requests, dispensers were onboarded to the XATP application and were required to authenticate their identities with supporting documentation. PIC documentation was routed to an external validator, as shown in Fig. 1. Conversely, POA documentation was sent to the PIC, as only the PIC has the authority to authenticate and confer Power of Attorney (POA) to other pharmacy employees (19). In this way, the PIC and POA form a single pharmacy workgroup.

**Fig. 2:**
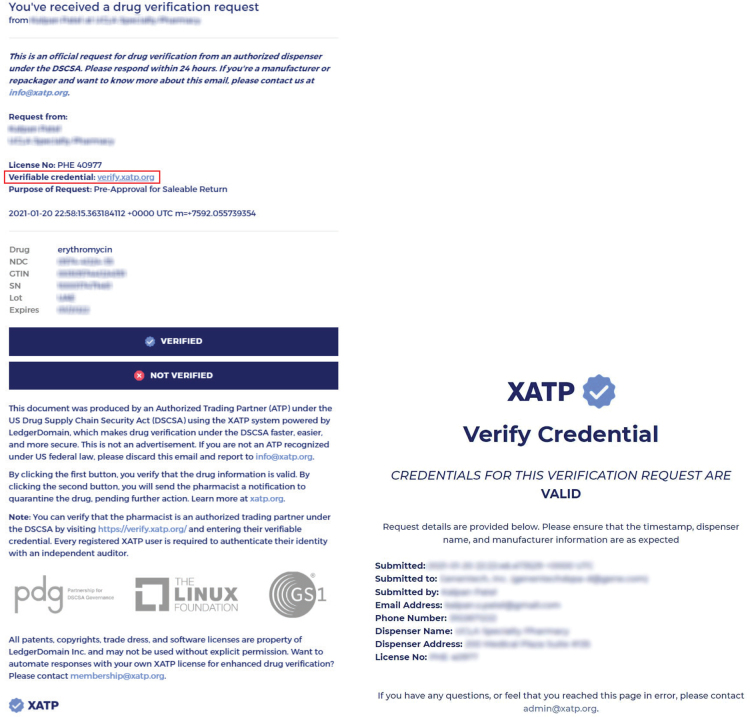
(Left) An enhanced verification request received by a manufacturer, with a verifiable credential link highlighted in red. (Right) An identity credential verification hosted at a secure web endpoint.

During the course of testing, dispensers submitted drug verification requests to participating manufacturers, as well as to randomly selected manufacturers, based on drug packages selected randomly from pharmacy inventory by the pharmacist. Manufacturer users were able to respond to verification requests embedded in messages encompassing the verification request and the verifiable credential of the requestor. These messages took the form of emails, which could be independently verified by the responder, as shown in Fig. 2.

### Round 1

In the first round of testing, 30 genuine drug packages with barcodes were used to test package and medication guide (20) accuracy, and 39 synthetic barcodes were used in combination with pilot manufacturer emails (sent to non-production email addresses at the manufacturer) to test expiration flagging and routing of verification requests (Table 2).

**Table 2:** Round 1 test objectives, methods, and results

Objective	Data source	Method	Results
Test package and medication guide accuracy	30 genuine drug packages, selected at random from the pharmacy	Three dispensers each scanning 10 packages, confirming package and med guide accuracy	100% (30/30) success rate in accurate drug scanning 97% (29/30) success rate in looking up electronic drug package medication guides
Test routing of synthetic verification requests (dispenser side)	39 synthetic drug packages, consisting of two different manufacturers and six different drugs (including 12 expired drugs)	Two dispensers scanning synthetic drug packages and observing app behavior	100% (39/39) success rate in accurate drug scanning 100% (39/39) success rate in submitting drug verification requests 100% (39/39) success rate in receiving drug verification status updates 100% (39/39) success rate in identifying expired drugs
Test routing of synthetic verification requests (manufacturer side)	Emails generated from scanning of 39 drug packages and synthetic serialization data records	Two manufacturers receiving and manually reviewing extracted synthetic barcode data against synthetic serialization data records	100% (39/39) success rate in receiving verification requests 86% (32/39) accuracy in drug verification matching against synthetic serialization data records 100% (39/39) success rate in responding to verification requests

Prior to the test, participating manufacturers were provided with synthetic serialization data records (collectively totaling 1,008 records), and dispensers were provided with 39 synthetic barcodes. Twenty-three of these barcodes corresponded to records in the databases (and could be ‘verified’), while 16 did not (and were notionally ‘counterfeit’). It should be noted that no actual counterfeits were uncovered through the course of testing.

As shown in the table, the authors observed a 100% success rate in submitting, receiving, and responding to drug verification requests on the part of both dispensers and manufacturers. Owing to human factors, 32 of 39 verifications (86%) were successful, as there were four false negatives and three false positives.

### Round 2

**Fig. 3:**
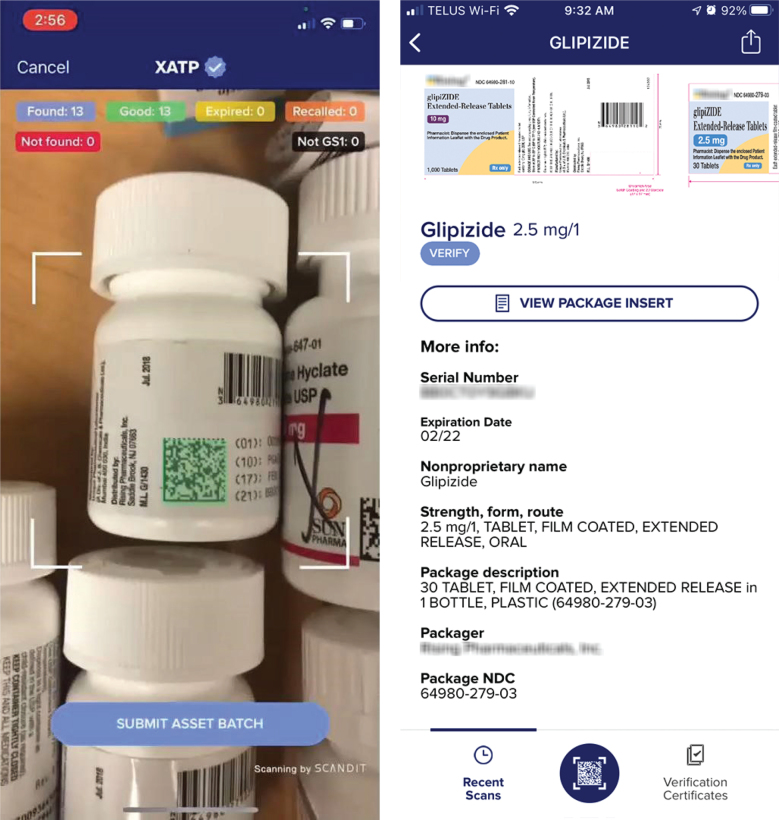
(Left) Genuine barcodes being scanned at the pharmacy. (Right) The scanned drug in the XATP app.

The second round of testing focused on testing enhanced verification with genuine drugs (Fig. 3), with both participating and randomly selected manufacturers (Table 3).

**Table 3:** Round 2 test objectives, methods, and results

Objective	Data source	Method	Results
Test routing of genuine verification requests to participating manufacturers	37 genuine drug packages originating from participating manufacturers, selected from the pharmacy	Two dispensers scanning synthetic drug packages and observing app behavior	100% (37/37) success rate in accurate drug scanning
	Emails generated from scanning of 37 drug packages and genuine serialization data records	Three manufacturers receiving and manually reviewing extracted genuine barcode data against genuine serialization data records	100% (37/37) success rate in receiving verification requests 100% (37/37) success rate in responding to verification requests
Test recall flagging functionality	Five synthetic drug packages with barcodes corresponding to FDA recalls	One dispenser scanning synthetic drug packages	100% (5/5) success rate in identifying the recalled product
Test routing of genuine verification requests to randomly selected manufacturers	27 genuine drug packages selected at random from the pharmacy (resulting in 11 randomly selected manufacturers)	One dispenser scanning genuine drug packages and sending verification requests	100% (27/27) success rate in accurate drug scanning 88% (23/27) success rate in submitting drug verification requests
Test verifiable credential	Two verifiable credentials included in emails to manufacturers	Two manufacturers authenticating emailed requests	100% (2/2) verifiable credentials successfully authenticated

During this round, 64 packages were scanned, in total. As three NDCs comprising four products could not be matched to manufacturer POCs, 60 verification requests were submitted and 60 emails were confirmed to have been sent. Overall, the authors observed a 94% success rate in submitting drug verification requests, with the 6% attributed to smaller manufacturers outside the global top 1,000 pharmaceutical companies (21).

### Post-round evaluations

Following the completion of Round 2, the authors evaluated the independent verifiability of the drug verification requests, as well as the Product Verification Certificates generated by the pharmacy workgroup. They also received feedback from participating and selected manufacturers.

For the drug verification requests, participating manufacturers tested and successfully authenticated the associated identity credential shown in Fig. 2. This credential, which is linked in the email and is hosted at a secure web endpoint, enables responders to ensure that an email from a requestor is genuine (and not a counterfeiter attempting to gather sensitive information). One of the manufacturers reported that emails had been routed to the incorrect contact. The dispensers and another manufacturer encountered difficulties with the emails, which was found to be the result of link wrapping services executed by their organizations’ IT security policies. This required resubmission of verification requests and pointed to the need for domain whitelisting to ensure secure interoperability.

**Fig. 4:**
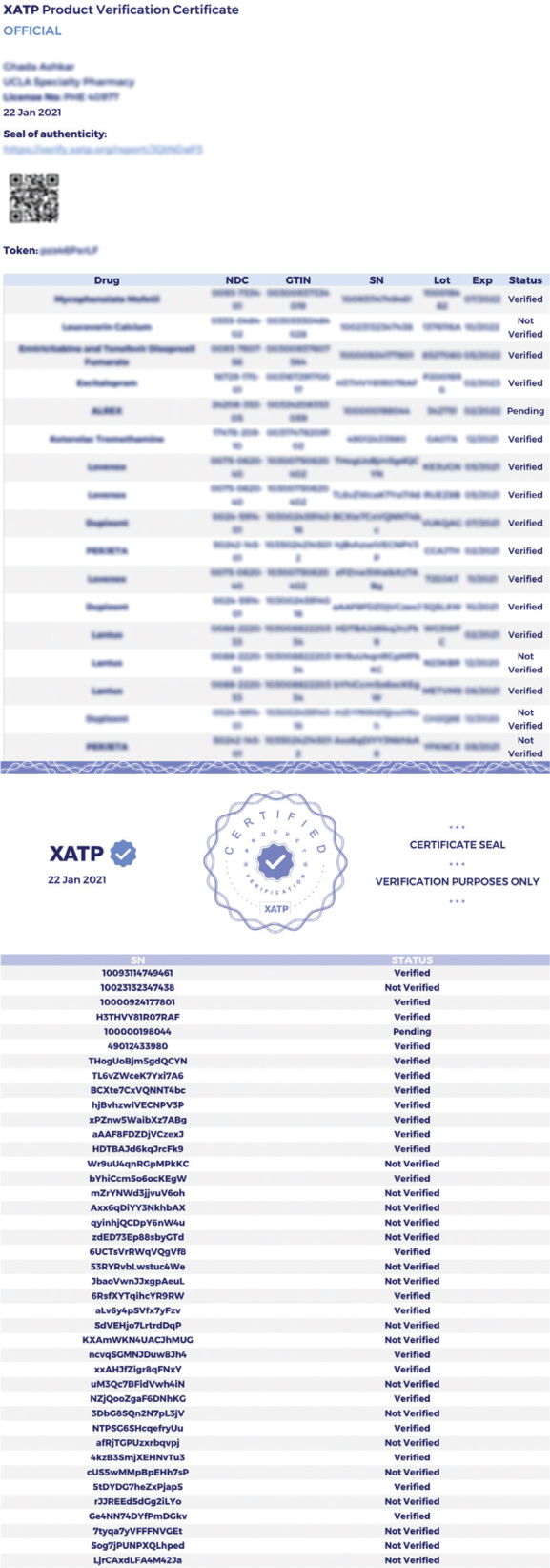
(Left) The first page of a Product Verification Certificate generated by a dispenser. (Right) The first page of the corresponding Certificate Seal. Note that in both cases the ‘not verified’ statuses refer to synthetic barcodes tested during Round 1.

As noted previously, dispensers have the ability to generate Product Verification Certificates that can be shared with, and independently authenticated by, other ATPs. This provides the name, GTIN, NDC, serial number, lot number, expiration date, and verification status for each unit listed. As shown in Fig. 4, each Certificate also bears a URL and access token to a web portal where the user can access its corresponding Certificate Seal, which can be used to authenticate the Certificate. This process can facilitate a verifiable record to show that drugs being received by a third party have been verified and may be sold.

The Certificate Seal includes a list of drugs and their verification statuses, but contains only truncated drug information (22). Certificate holders have the ability to access the Seal and compare it with the data on the Certificate, making it possible to ensure that their Certificate is genuine and untampered. The authors employed Certificate and Seal for analysis of the study results, and found that the former could be successfully authenticated by the latter.

After the round, five of the 14 manufacturers contacted sent verifications in a timely manner: the three participating manufacturers, another global top 15 manufacturer, and a specialty manufacturer. The authors were also contacted by two randomly selected manufacturers requesting additional information regarding the verification requests. One was based outside the United States and directed dispensers to its US subsidiary; the other indicated that requests are preferably routed through a proprietary VRS (a functionality common for wholesale distributors in verification of saleable returns, but requiring expansion to the broader ATP community).

## Discussion and conclusions

In this study, the authors tested a framework for ATP authentication, enhanced verification, and saleable returns documentation in a real-world setting using both genuine and synthetic drug packages to test positive and negative verification workflows. Synthetic recalls and expired drugs were successfully flagged, and 94% of genuine drug verification requests were successfully delivered to participating and randomly selected manufacturers. Each manufacturer was only able to access his or her own verification requests.

Once the identity and ATP status of the PIC were successfully authenticated by an external validator, the PIC, in turn, was able to authenticate POAs, forming a pharmacy workgroup. Within the workgroup, the PIC and POAs shared a common pool of scanned barcodes and Product Verification Certificates, and were able to see verification statuses updated in real time. Each dispenser user held his or her own locally encrypted private key, which was generated in concert with signup.

Outside the pharmacy workgroup, other ATPs were proven able to interact with the XATP framework using email clients and web browsers, without the need to install new software or create accounts. Manufacturers received verification requests signed with verifiable credentials, which could be independently authenticated, and were able to respond with the click of a button. Dispensers generated supporting documentation in the form of Product Verification Certificates, which were also independently verifiable.

By directly addressing the need for ATPs under the DSCSA to have a secure, reliable, and seamless remote method for digital IDs, in combination with commercial off-the-shelf mobile phones (23), the framework outlined in this study allowed for ‘day zero’ interoperability between manufacturers and dispensers with no prior business relationship. All of the major pharmaceutical companies that were contacted sent verifications in a timely manner.

**Fig. 5:**
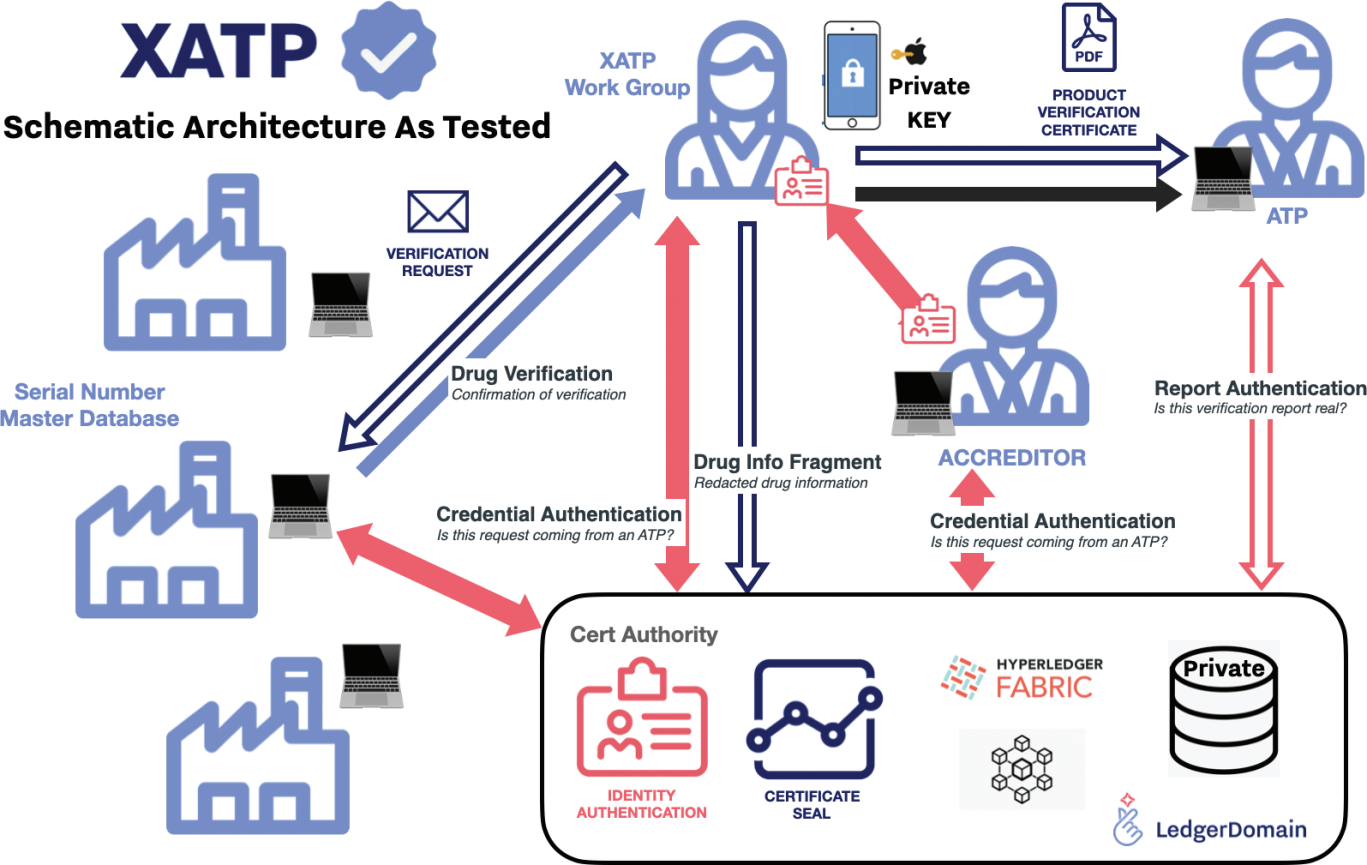
The framework architecture as tested in this study.

While the study framework involved human-in-the-loop workflows (Fig. 5), the authors anticipate that scaled implementations can be partially or fully automated through existing integration to manufacturer serialization data sources. Once provisioned with agents to test verifiable credentials, machine-to-machine connections between the framework and manufacturer relational databases would manage identity credentials and automatically respond to and sign verification requests. Interoperability with other frameworks could be achieved with an API that enables third parties to make verification requests.

Critically, by ensuring that private keys are held by stakeholders as part of a robust identity system, the XATP framework mitigates the single-point-of-attack risks of legacy providers, where the keys to responder databases are often held and pooled. Much like a janitor’s keyring, which grants access to every room in a building, a single security breach in such systems might allow attackers to hijack other identities, create false identities, or gain access to confidential data (24, 25, 26, 27). By allowing for passwordless access closely associated with a device, XATP also sidesteps the risks associated with passwords, including sharing, leaks, and sharing passwords across multiple services (28, 29, 30). The XATP framework thus mitigates the risk for stakeholders to rapidly attain compliance with DSCSA obligations, such as drug verification, and sets a path for greater interoperability leveraging verifiable credentials (31) in the broader healthcare community.
